# Digital gene expression analysis of gene expression differences within *Brassica* diploids and allopolyploids

**DOI:** 10.1186/s12870-015-0417-5

**Published:** 2015-01-27

**Authors:** Jinjin Jiang, Yue Wang, Bao Zhu, Tingting Fang, Yujie Fang, Youping Wang

**Affiliations:** Jiangsu Provincial Key Laboratory of Crop Genetics and Physiology, Yangzhou University, Yangzhou, 225009 China

**Keywords:** *Brassica* spp, Polyploidization, Sequencing, Digital gene expression (DGE)

## Abstract

**Background:**

*Brassica* includes many successfully cultivated crop species of polyploid origin, either by ancestral genome triplication or by hybridization between two diploid progenitors, displaying complex repetitive sequences and transposons. The U’s triangle, which consists of three diploids and three amphidiploids, is optimal for the analysis of complicated genomes after polyploidization. Next-generation sequencing enables the transcriptome profiling of polyploids on a global scale.

**Results:**

We examined the gene expression patterns of three diploids (*Brassica rapa*, *B. nigra*, and *B. oleracea*) and three amphidiploids (*B. napus*, *B. juncea*, and *B. carinata*) via digital gene expression analysis. In total, the libraries generated between 5.7 and 6.1 million raw reads, and the clean tags of each library were mapped to 18547–21995 genes of *B. rapa* genome. The unambiguous tag-mapped genes in the libraries were compared. Moreover, the majority of differentially expressed genes (DEGs) were explored among diploids as well as between diploids and amphidiploids. Gene ontological analysis was performed to functionally categorize these DEGs into different classes. The Kyoto Encyclopedia of Genes and Genomes analysis was performed to assign these DEGs into approximately 120 pathways, among which the metabolic pathway, biosynthesis of secondary metabolites, and peroxisomal pathway were enriched. The non-additive genes in *Brassica* amphidiploids were analyzed, and the results indicated that orthologous genes in polyploids are frequently expressed in a non-additive pattern. Methyltransferase genes showed differential expression pattern in *Brassica* species.

**Conclusion:**

Our results provided an understanding of the transcriptome complexity of natural *Brassica* species. The gene expression changes in diploids and allopolyploids may help elucidate the morphological and physiological differences among *Brassica* species.

**Electronic supplementary material:**

The online version of this article (doi:10.1186/s12870-015-0417-5) contains supplementary material, which is available to authorized users.

## Background

Polyploidy is an important factor in the evolution of many plants and has attracted considerable scientific attention for a long period of time. Many important economical crops are of polyploid origin, including wheat, cotton, and rapeseed [1]. *Cruciferae* includes the model species *Arabidopsis thaliana* and the economically important *Brassica* crops. These important crops include three diploid *Brassica* species, namely, *B. rapa* (AA, 2*n* = 20; Chinese cabbage, turnip, turnip rape), *B. nigra* (BB, 2*n* = 16; black mustard), and *B. oleracea* (CC, 2*n* = 18; cauliflower, broccoli, kale), and three allopolyploids spontaneously derived from pairwise hybridization of the diploids, which are *B. napus* (AACC, 2*n* = 38; oilseed rape, swede), *B. juncea* (AABB, 2*n* = 36; abyssinian or Ethiopian mustard), and *B. carinata* (BBCC, 2*n* = 34; Indian or brown mustard) [2]. Lysak et al. (2005) confirmed the chromosome triplication history of *Brassica* that corresponds to that of *Arabidopsis* [3]. Cheung et al. (2009) found that the divergence of *Arabidopsis* and *Brassica* lineage was approximately 17 Mya [4], and the replicated *Brassica* subgenomes (probably the divergence of A/C from B genome) was diverged by 14.3 Mya [4]. In addition, the A and C genomes were estimated with 3.7 Mya divergence. Woodhouse et al. (2014) stated that the *B. rapa* lineage underwent a whole-genome triplication of 5–9 Mya [5]. For the allopolyploids, *B. napus* probably arose from the natural hybridization of A and C genomes around 10,000 years ago. However, when the hybridization between A and B genomes and between B and C genomes happened is still unclear. The precise ancestors of *B. napus*, *B. juncea*, and *B. carinata* are not yet identified [6]. The duplication of gene segments reported on *Brassica* is explained as frequent loss, remote genome duplication, or unbalanced homologous recombination [7]. During the divergence of *Brassica* species, the sub-functionalization and/or neo-functionalization of these paralogs coupled with novel gene interactions contribute significantly to genome evolution [8]. Moreover, genetic mapping and sequencing analysis substantiate the triplication hypothesis of diploid *Brassica* genomes [9-12]. The comparative mapping of *Brassica* by using genetic markers has successfully revealed homologous rearrangements, translocations, and fusions that are crucial to the diversification of the A, B, and C genomes from *A. thaliana* [13-15].

Many linkage maps and karyotype analysis have identified extensive collinearity and genomic polymorphisms among *Brassica* genomes. Given the complexity of the gene copy number and syntenic conservation caused by polyploidization, *Brassica* genomes are difficult to study [16,17]. Identifying the genes related to specific traits based on the linkage maps is also challenging because of the complexity of the homologs and paralogs in polyploidy genomes [15,18]. Profiling arrays of *A. thaliana* are useful in the transcriptome analysis of *Brassica* [6]. However, *A. thaliana*-based microarrays lack the resolution of *Brassica* specific genes and paralogous genes. Furthermore, *Brassica* microarrays were developed to confirm *Brassica*-specific expressed genes [19]. Identifying different homologous copies of *Brassica* sequences is challenging for microarray expression analysis [20]. Next-generation sequencing is an optimal method for genomic and transcriptomic studies and provides opportunities for polyploidy studies and enables the extensive genome profiling of crops with complex genomes, such as soybean, potato, tomato, cotton, maize, and common bean [21-26]. This technology also promotes sequencing analysis in *Brassica*; the genome sequence of *B. rapa* has already been released and annotated [12]. The genome sequencing of *B. oleracea*, *B. nigra*, and *B. napus* is still in progress. However, the genome sequences of *B. oleracea* are available in the Basic Local Alignment Search Tool in *Brassica* database (www.brassica.info). The transcriptome profiling of *B. napus* has been analyzed via RNA sequencing [27-29]. This information is valuable for the investigation of *Brassica* genome evolution. Many technologies have been applied to quantify transcript abundance, including microarray, serial analysis of gene expression, digital gene expression (DGE), and RNA-seq. DGE and RNA-seq have been widely used to identify the molecular information of plant transcriptome and gene expression variation between comparable samples. DGE, as a well-known technique suitable to directly quantify transcript abundance counts, is optimized over RNA-seq because of its cost efficiency. RNA-seq is a flexible approach that can detect full-transcript sequence, alternative splicing, exon boundaries, and transcript abundance. However, each transcript in RNA-seq can be mapped multiple times, and the sequencing depth of RNA-seq is correlated with but is not equal to transcript abundance. Each read in DGE is expected with a sole hit on an RNA molecule. Therefore, DGE is better to represent rare transcripts or exclude transcripts with less interest than RNA-seq [30].

Many studies have analyzed the genomic and phenotypic changes in synthesized *Brassica*, particularly *B. napus* and hexaploid *Brassica* [31-33]. However, limited information is available for the natural species of *Brassica*. In the present research, we performed DGE analysis on three diploid *Brassica* species (*B. rapa*, *B. nigra*, and *B. oleracea*) and three allopolyploids (*B. napus*, *B. juncea*, and *B. carinata*) to determine the transcriptome changes after natural polyploidization. The expression profile of the genes in the six *Brassica* species was reported, and the multiple gene expression differences were observed. Differentially expressed genes (DEGs) are involved in a wide range of stress resistance and development processes. This study is the first transcriptomic research that identifies DEGs and the pathways involved in the natural polyploidization of the six *Brassica* species.

## Results

### DGE profile

**Figure 1:**
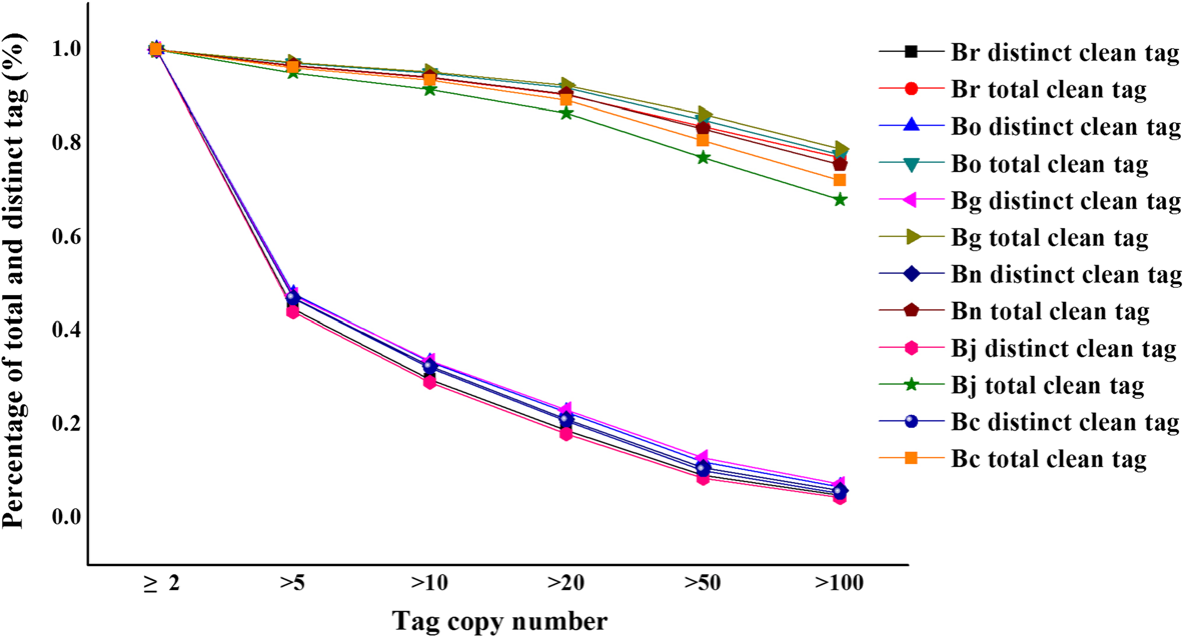
**Distribution of total tag and distinct tag counts over different tag abundance categories from the six libraries.**

This research investigates the transcriptome profiling of diploids and spontaneous allopolyploids in *Brassica* by performing DGE analysis on the seeding stage of the six *Brassica* species, namely, *B. rapa* (Br), *B. nigra* (Bg), *B. oleracea* (Bo), *B. napus* (Bn), *B. juncea* (Bj), and *B. carinata* (Bc). DGE libraries from the leaves of four-week-old plants were generated and sequenced by an Illumina technology. The sequence data are available from the GEO repository with an accession number of GSE43246. The statistics of the DGE tags are shown in Table 1. Approximately six million raw tags were generated for each library. Clean tags were obtained after removing the low-quality sequences and adaptor sequences from the raw data. 6178564, 5881618, 6059222, 5964594, 6076830, and 5795234 clean tags were obtained in Br, Bg, Bo, Bn, Bj, and Bc, respectively. Unambiguous tags (tags that were uniquely matched to one gene of reference genome with no more than one mismatch) were counted and normalized to TPM to evaluate the gene expression level. To evaluate the normality of the DGE data, the distribution of the total tags and distinct clean tags (tags with specific nucleotide sequence) over different tag copy numbers was analyzed. The distribution of the tag expression was similar for each library. Moreover, the distribution of clean tags in the six libraries showed that most of the tags are from highly expressed genes (Figure 1, Additional files 1 and 2). The percentage of distinct tags with high counts dropped dramatically, and the distinct tags with more than 100 copies accounted for less than 8%. However, more than 67% of the total clean tags accounted for more than 100 copies in each library. By contrast, more than 43% of the distinct clean tags had copy numbers between two and five, which represented approximately 96% of the total number of clean tags. Generally, a small number of categories of mRNA showed high abundance, whereas the other majority had a quite low expression level. This finding indicates that only a small number of mRNAs are expressed at high abundance, and majority of them are expressed at very low levels [34].

**Table 1:** **Statistics of categorization and abundance of DGE tags**

**Summary**		***B. rapa***	***B. nigra***	***B. oleracea***	***B. napus***	***B. juncea***	***B. carinata***
Raw Tag	Total	6178564	5881618	6059222	5964594	6076830	5795234
Raw Tag	Distinct Tag	293575	214427	243895	269285	400134	278768
Clean Tag	Total number	6018254	5772449	5930726	5823113	5858527	5657697
Clean Tag	Distinct Tag number	133499	106552	116771	128967	181965	142281
Tag Mapping to Gene	Total number	1964909	1990442	1747843	2253347	1857572	1915305
Tag Mapping to Gene	Distinct Tag number	44267	30413	36220	45358	56289	40425
Unambiguous Tag Mapping to Gene	Total number	1679848	1635594	1475050	1924944	1531974	1594991
Unambiguous Tag Mapping to Gene	Total% of clean tag	27.91%	28.33%	24.87%	33.06%	26.15%	28.19%
Unambiguous Tag Mapping to Gene	Distinct Tag number	39414	26114	31933	40561	49892	35285
Unambiguous Tag Mapping to Gene	Distinct Tag% of clean tag	29.52%	24.51%	27.35%	31.45%	27.42%	24.80%
Tag-mapped Genes	number	19023	16687	18547	19955	21995	19436
Tag-mapped Genes	% of ref genes	46.20%	40.53%	45.05%	48.47%	53.42%	47.20%
Unambiguous Tag-mapped Genes	number	16574	13867	15970	17448	19424	16645
Unambiguous Tag-mapped Genes	% of ref genes	40.25%	33.68%	38.79%	42.38%	47.18%	40.43%
Mapping to Genome	Total number	2437918	1147106	2105332	2164464	2047451	1462061
Mapping to Genome	Total% of clean tag	40.51%	19.87%	35.50%	37.17%	34.95%	25.84%
Mapping to Genome	Distinct Tag number	44076	15159	30703	40689	50304	29547
Mapping to Genome	Distinct Tag% of clean tag	33.02%	14.23%	26.29%	31.55%	27.64%	20.77%
Unknown Tag	Total number	1615427	2634901	2077551	1405302	1953504	2280331
Unknown Tag	Total% of clean tag	26.84%	45.65%	35.03%	24.13%	33.34%	40.30%
Unknown Tag	Distinct Tag number	45156	60980	49848	42920	75372	72309
Unknown Tag	Distinct Tag% of clean tag	33.82%	57.23%	42.69%	33.28%	41.42%	50.82%

Clean tags are tags after filtering low-quality tags from raw data. Distinct tags are different tags and unambiguous tags are the remaining clean tags after removing tags mapped to more than one locus of reference genome.

The clean tags were then mapped onto the *B. rapa* genome with a maximum of one base-pair mismatch [12]. Table 1 shows that the 1964909, 1990442, 1747843, 2253347, 1857572, and 1915305 tags in Br, Bg, Bo, Bn, Bj, and Bc were mapped to *B. rapa* genome, respectively. Statistical analysis of clean tag alignment was conducted, including the analysis of total clean tags and distinct clean tags (Additional files 2 and 3). More than 54% of the total clean tags and 42% of the distinct clean tags in each library were mapped onto the *B. rapa* genome. However, the tags mapped in the DGE library of Bg and Bc were lower than those in the other four libraries, which might be due to the divergence of the B genome to the A/C genome. Moreover, the tag mapping onto the *B. rapa* genome generated 19023 tag-mapped genes for Br, 16687 for Bg, 18547 for Bo, 19955 for Bn, 21995 for Bj, and 19436 for Bc. In total, approximately 61% of the genes in the *B. rapa* genome (25298 genes) could be mapped with unique tags (Additional file 4). Furthermore, we mapped all the clean tags of each DGE library to the genome of *A. thaliana*, and the summary and details of the mapping result are listed in Additional file 5. Only approximately 47% of *A. thaliana* genes (19557 genes) were successfully mapped, and the percent of unambiguous tag-mapped genes in *A. thaliana* is much lower than in *B. rapa*. The number of DGE tags in each library that well matched with *Arabidopsis* genome is also lower than that mapped to *B. rapa*. The difference in mapping rate is in accordance with the prediction that the A, B, and C genomes of *Brassica* diverged after the divergence of *Arabidopsis* and *Brassica* lineages [6]. Thus, we chose the mapping information that used *B. rapa* as reference for further analysis. Saturation analysis was performed to check if the number of detected genes increased with sequencing amount. The result showed that the number of detected genes stopped increasing when the number of reads reached 2 million (Additional file 6). The distribution of the ratio of distinct tag copy numbers in each pair of libraries was analyzed. More than 90% of the distinct tags had ratios up to five folds (Additional file 7).

### DEGs in *Brassica* diploids

**Figure 2:**
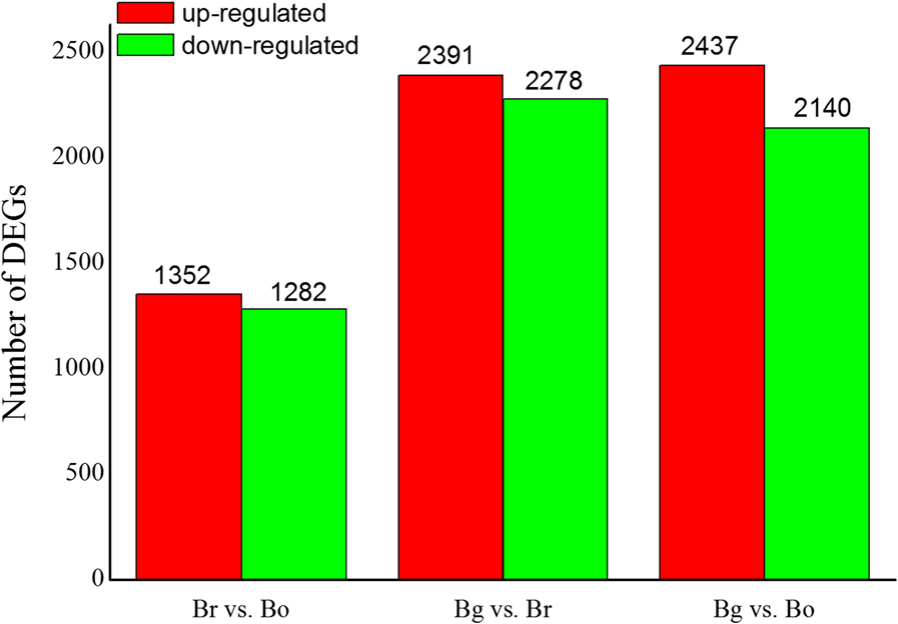
**Number of differentially expressed genes in each comparison of** ***Brassica*** **diploids.** The numbers of up-regulated (in red) and down-regulated genes (in green) are presented. Br, Bg and Bo are abbreviations of *B. rapa*, *B. nigra* and *B. oleracea*, respectively.

The DEGs in *Brassica* diploids (Br, Bg, and Bo) were compared (Br vs. Bo, Bg vs. Br, Bg vs. Bo, where A was the control group and B was the experimental group in “A vs. B”) to analyze the gene expression variations (Figure 2 and Additional file 8). A comparison of Br and Bo showed that 1352 and 1282 DEGs were significantly up-regulated and down-regulated, respectively. By contrast, 2278 DEGs were down-regulated and 2391 DEGs were up-regulated in Br compared with Bg.

**Figure 3:**
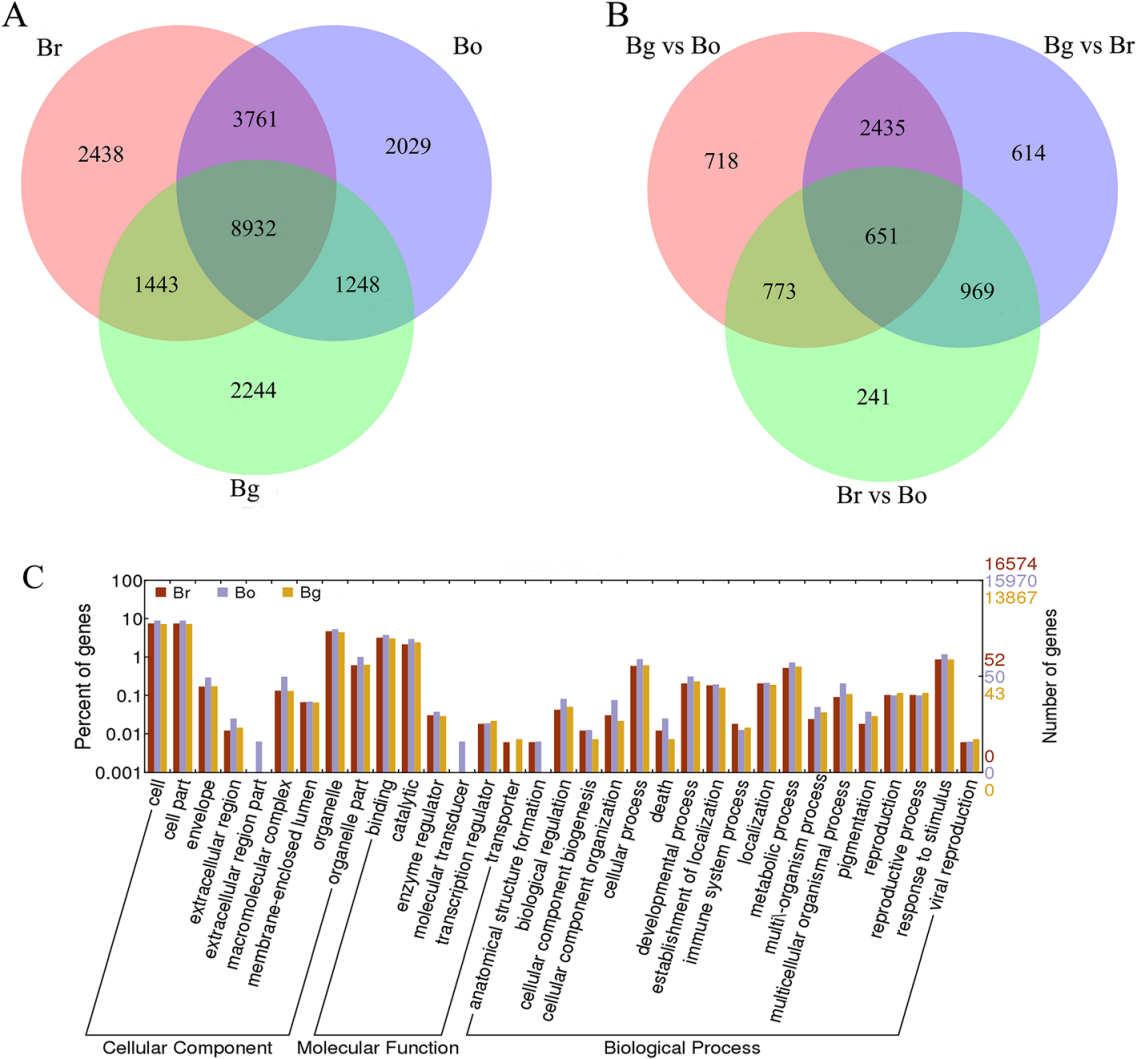
**Distribution of expressed mRNAs in** ***Brassica*** **diploids. A.** Venn diagram of genes expressed in Br, Bg and Bo. **B.** Venn diagram of unique expressed genes between pairwise of Br, Bg and Bo. **C.** GO classification of genes in Br, Bg and Bo.

Moreover, 2140 DEGs were down-regulated and 2437 DEGs were up-regulated in Bo compared with Bg. The number of DEGs in Bg compared with Br/Bo was more than Br vs. Bo, which indicates that the A and C genomes of *Brassica* were closer than the B genome. Among the 20 most abundantly expressed genes that were up-regulated or down-regulated in the pair comparison of the three diploids (Additional file 8), Bra015187, Bra026992, Bra017452, Bra029372, Bra028406, Bra017112, Bra036352, Bra000377, and Bra016934 were up-regulated in Bg compared with Br/Bo. Moreover, Bra023103, Bra011285, Bra014371, Bra031070, Bra028805, and Bra006083 were down-regulated in Bg compared with Br/Bo. Most DEGs between Br and Bo differed from those between Br and Bg as well as between Bo and Bg. Figure 3A shows the distribution of the genes commonly expressed in Br, Bg, and Bo, and 8932 genes were co-expressed in the three diploid libraries apart from the DEGs. A second filter of expression differences (at least twofold or greater) was performed in the pairwise comparisons to reduce the total number of significant changes. This analysis revealed 6401 significantly expressed genes, such as Br vs. Bg = 4669, Br vs. Bo = 2634, and Bg vs. Bo = 4577 (Figure 3B). The functional significance of the genes expressed in each library was examined, and the GO analysis results are shown in Figure 3C. The well-annotated gene sequences were assigned to 33 functional groups of the three main GO categories (cellular component, molecular function, and biological process). The results showed that DGEs were primarily involved in the cell and organelle, in the binding, catalytic, cellular, and metabolic process, as well as in response to stimulus. Two specific processes, the extracellular region part and the molecular transducer, were unique to Bo. However, Bo lacked a transporter, and Bg lacked anatomical structure formation.

### DEGs among allopolyploids and ancestral diploid progenitors

**Figure 4:**
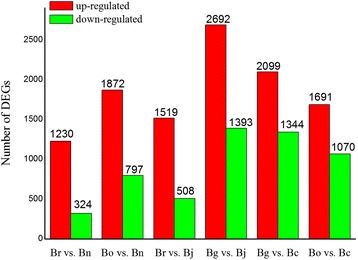
**Number of differentially expressed genes in comparison of diploids and amphidiploids.** The numbers of up-regulated (in red) and down-regulated genes (in green) are presented. Br, Bg, Bo, Bn, Bj and Bc are abbreviations of *B. rapa*, *B. nigra*, *B. oleracea*, *B. napus*, *B. juncea* and *B. carinata*, respectively.

**Figure 5:**
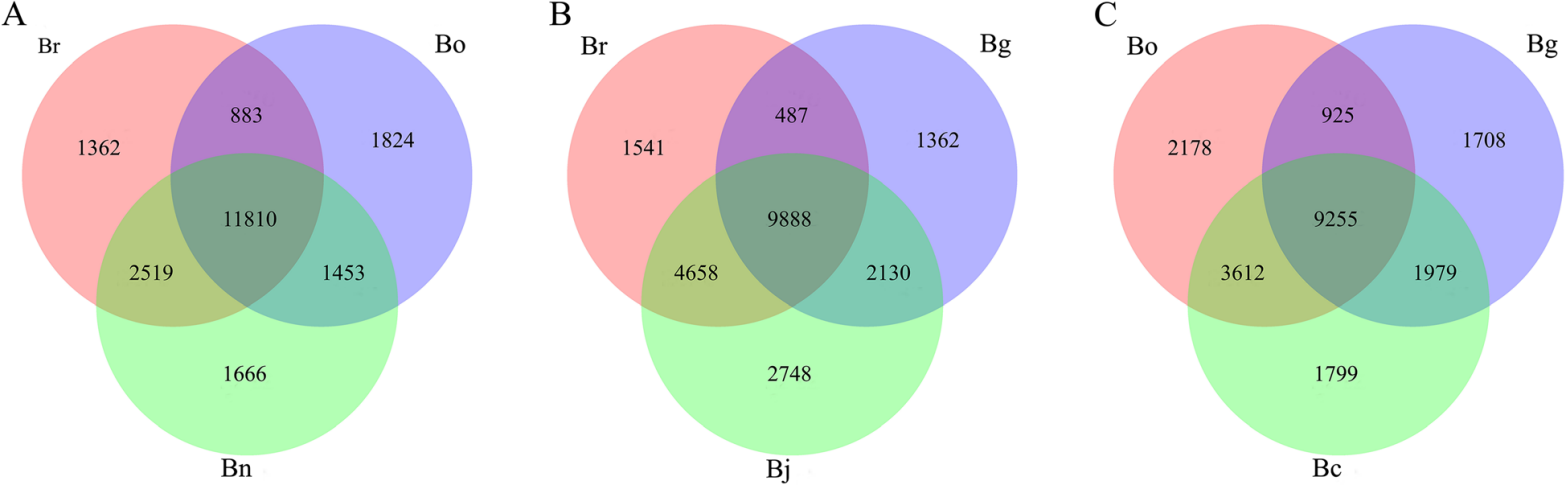
**Distribution of the genes commonly and specifically expressed in diploids and amphidiploids. A.** Venn diagram of genes expressed in Br, Bo and Bn. **B.** Venn diagram of genes expressed in Br, Bg and Bj. **C.** Venn diagram of genes expressed in Bg, Bo and Bc.

**Figure 6:**
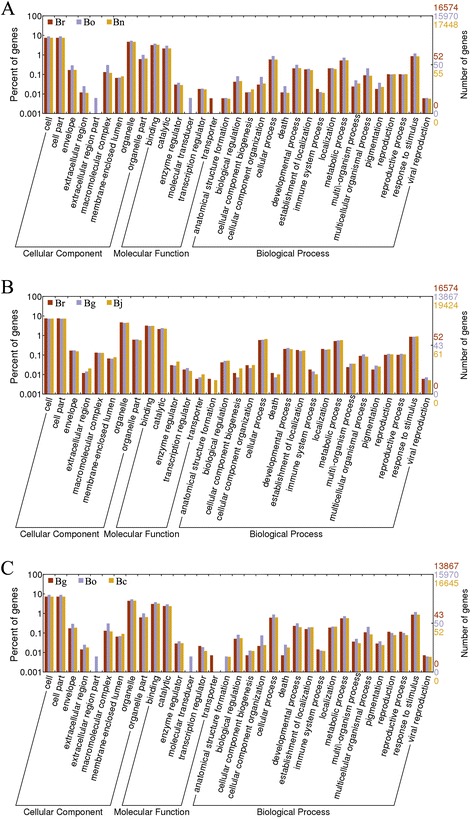
**GO classification of genes in diploids and amphidiploids. A.** GO classification of genes expressed in Br, Bo and Bn. **B.** GO classification of genes expressed in Br, Bg and Bj. **C.** GO classification of genes expressed in Bg, Bo and Bc.

To identify the DEGs in allopolyploids and ancient diploid progenitors, the DGE profiles of Br vs. Bn, Bo vs. Bn, Br vs. Bj, Bg vs. Bj, Bg vs. Bc, and Bo vs. Bc were compared to analyze the gene expression variations after natural polyploidization (Figure 4 and Additional file 8). The results showed that 1230 DEGs were up-regulated and 324 DEGs were down-regulated in Bn compared with Br, whereas 1872 DEGs were up-regulated and 797 DEGs were down-regulated in Bn compared with Bo. After natural polyploidization, 1519 DEGs were induced in Bj compared with Br, whereas 508 DEGs were down-regulated. Moreover, 2692 DEGs were induced in Bj compared with Bg, whereas 1393 DEGs were down-regulated. With regard to Bc, 2099 DEGs were up-regulated and 1344 were down-regulated compared with Bg, and 1691 DEGs were up-regulated and 1070 were down-regulated compared with Bg. The variations in the gene expression among the diploids and amphidiploids are essential to the diversity of phenotype, growth, and production. The 20 most abundantly expressed genes that were up-regulated or down-regulated in the pair comparison of amphidiploids and diploids are listed in Additional file 8. The distribution of the genes that were commonly and uniquely expressed in amphidiploid and its ancestral diploids is shown in Figure 5. The results further show that 11810 genes were conserved in Br, Bo, and Bn, whereas 1362, 1666, and 1824 genes were specifically expressed in Br, Bo, and Bn, respectively (Figure 5A). A similar pattern was observed when Bj was compared with Br/Bg (Figure 5B) and Bc with Bg/Bo (Figure 5C). The expressional differences of species-specific genes might be caused by the genome interaction during natural polyploidization. The GO pattern of the genes in amphidiploid and ancestral diploids is shown in Figure 6. Based on Figure 6A, the numbers of DGEs in the envelope, extracellular region, macromolecular complex, biological regulation, cellular component biogenesis, death, multicellular organism process, and pigmentation were different in Br, Bo, and Bn. GOs of molecular transducer was found in Bo only. Apparent GO difference was observed among Br, Bg, and Bj (Figure 6C). As shown in Figure 6C, GOs of transporter were found in Bg only, and anatomical structure formation was not present in Bg.

### Functional annotation of DEGs

Pathway enrichment analysis was performed on the expressed transcripts of the six DGE libraries. This analysis was performed by mapping all annotated genes in the KEGG database to search for significantly enriched genes involved in the metabolic or signal transduction pathways (Additional file 9). Among the genes with KEGG annotation, DEGs were identified in Bn compared with Br. In total, 894 DEGs were assigned to 109 KEGG pathways, and 13 of these pathways were significantly enriched with *Q* values ≤ 0.05 (red border region). The enriched pathways include metabolic, biosynthesis of secondary metabolites, and peroxisome. A similiar pathway enrichment was discovered in pair comparison of other libraries (Bo vs. Bn, Br vs. Bj, Bg vs. Bj, Bg vs. Bc, and Bo vs. Bc). The 1562 DEGs identified in Bn vs. Bo were assigned to 122 KEGG pathways, 15 of which were significantly enriched. The 1171 DEGs identified in Bj vs. Br were assigned to 116 KEGG pathways, the 2373 DEGs identified in Bj vs. Bg were assigned to 121 pathways, the 1975 DEGs identified in Bc vs. Bg were assigned to 120 pathways, and the 1639 DEGs identified in Bc vs. Bo were assigned to 117 pathways. All these pathways are involved in the process of plant growth and stress reaction, which are important for the morphological and physiological differences among the *Brassica* species. The biosynthesis of unsaturated fatty acids, which was significantly enriched in Bo vs. Bn and Bg vs. Bc, explains the different fatty acid compositions in *Brassica* species [35,36]. The DEGs identified in the peroxisome pathway were related to the improved stress ability of Bn and Bj.

### Clustering of DEGs

**Figure 7:**
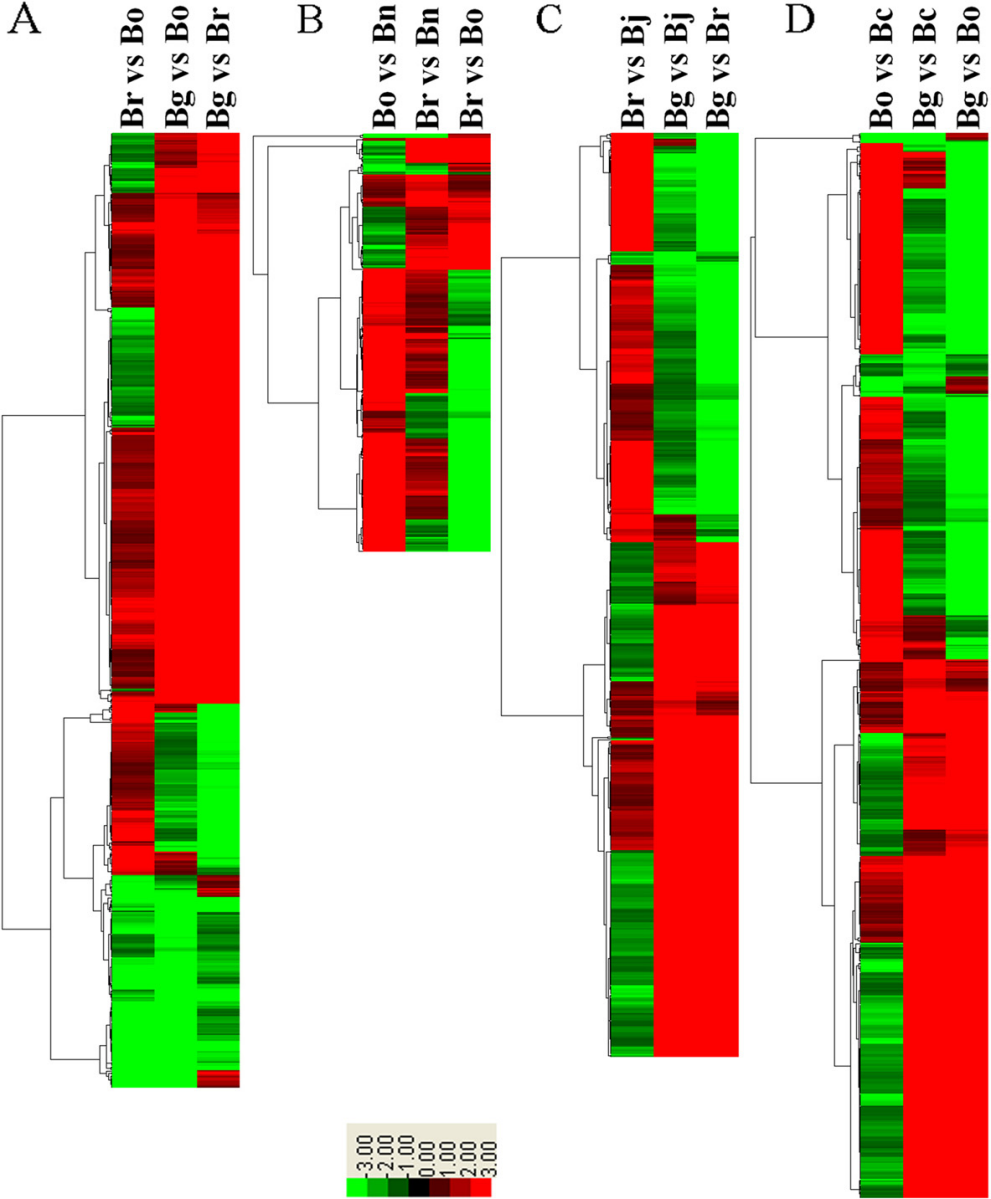
**Hierarchical cluster analysis of differentially expressed transcripts. A.** Clustering of genes expressed in diploids of *Brassica*. **B.** Clustering of genes expressed in Br, Bo and Bn. **C.** Clustering of genes expressed in Br, Bg and Bj. **D.** Clustering of genes expressed in Bg, Bo and Bc.

Hierarchical cluster analysis of the DEGs in Br, Bg, Bo, Bn, Bj, and Bc were performed to examine the similarity and diversity of gene expression (Additional file 4). All results were displayed by Java Treeview, where red and green represent the positive and negative values of gene expression, respectively. Generally, 651 genes with differential expression in Br, Bg, and Bo were clustered as DEG intersections (Figure 7A, Additional file 10). The comparison of Br, Bg, and Bo showed that 5417 DEGs were clustered as the union of DEGs (Additional file 11). Moreover, 285 DEGs in Bn, Br, and Bo were also clustered (Figure 7B and Additional file 9), and 3786 DEGs differentially expressed in Bn and Br/Bo are listed in Additional file 11. The 630 DEGs in Bj, Br, and Bg were also clustered (Figure 7C and Additional file 9), and 5590 DEGs differentially expressed in Bj and Br/Bg are listed in Additional file 11. In addition, 726 DEGs in Bc, Bg, and Bo were clustered (Figure 7D and Additional file 9), and 5264 DEGs differentially expressed in Bc and Bg/Bo are listed in Additional file 11. The functional categories of these DEGs showed similar enrichment patterns for each comparison, including categories of metabolism, development, cellular transport, and interaction with the environment (data not shown). Genes with binding function were enriched in each comparison, which is different from previous reports [32,33].

### Analysis of methyltransferase genes differentially expressed in *Brassica*

Epigenetic variation has an important function in the evolution of plants. DNA methylation is included in this variation and has received much attention in previous years. Proteins such as methyltransferase are considered important for plant methylation [37,38]. Thus, the putative methyltransferase and methylase genes from all DEGs in each comparison were filtered to understand the mechanism of the changes in DNA methylation in *Brassica* (Additional file 12). Two methyltransferase genes (Bra003928 and Bra020452) were differentially expressed in Br, Bg, and Bo, and 30 genes exhibited differential expression in Br vs. Bo/Bg vs. Bo/Bg vs. Br. One methyltransferase gene (Bra008507) was differentially expressed in Bn, Br, and Bo, and 23 genes exhibited differential expression in Br vs. Bn/Bo vs. Bn/Br vs. Bo. Five methyltransferase genes (Bra003396, Bra004391, Bra010977, Bra022603, and Bra024271) were differentially expressed in Bj, Br, and Bg, and 36 genes exhibited differential expression in Br vs. Bj/Bg vs. Bj/Bg vs. Br. Three methyltransferase genes (Bra003928, Bra004391, and Bra012494) were differentially expressed in Bc, Bg, and Bo, and 33 genes exhibited differential expression in Bg vs. Bc/Bo vs. Bc/Bg vs. Bo. The results showed that Bra003928 was significantly down-regulated in Br compared with Bg/Bo, which was up-regulated in Bn compared with Br and down-regulated in Bn compared with Bo. The expression of Bra003928 in Bj was higher than in Br and lower than in Bg. The expression of this methyltransferase gene in Bc was significantly up-regulated than in Bg and Bo. Moreover, Bra020452 was obviously down-regulated in Bo compared with Br/Bg. Different expression values were also examined in *Brassica* amphidiploids compared with their ancestral diploid parents. The methyltransferase gene was up-regulated in Bn compared with Br and Bo, which was also up-regulated in Bc compared with Bg and Bo. However, the expression value of Bra020452 in Bj was similar to that of Br and Bg.

### Non-additive genes expressed in *Brassica* amphidiploids

The expression value of genes in *Brassica* amphidiploids (Bn, Bj, and Bc) were compared with the relative mid-parent value (MPV) to identify the genes with differential expression pattern. Up to 19844 genes in Bn showed differences in expression from MPV, 9605 (48.4%) of these genes showed higher expression levels, whereas 10239 (51.6%) showed lower expressions than MPV. Among the non-additively expressed genes, 9519 (48%) genes were expressed at higher levels, whereas 10325 (52%) genes were expressed at lower levels in Br than in Bo (Table 2). This finding is similar to the data reported by Jiang et al. (2013) [32]. With regard to Bj, 20317 genes showed differences in expression from MPV, 11173 (55%) of these genes were expressed higher in Br than in Bg, and 9144 (45%) genes were expressed at lower levels. Moreover, 19921 genes in Bc showed differences in expression from MPV, 8189 (46.1%) of them were expressed higher in Bg than in Bo, whereas 10732 (53.9%) genes were expressed lower. Significantly more non-additive genes than additive genes in amphidiploids implied the complicated evolution history of *Brassica*. In this study, no standard RNA sample was included in library construction. Given that two samples often differ in the total number of transcripts per cell, the differences in transcriptome size, not just the differences in RNA yields during extraction, can introduce biases [39-41]. In addition, polyploidization of plant species is a complicated process that is unequal to simple duplication or combination of ancient genomes; fractionation of duplicated genes would result in both gene and genome preferences in stabilized *Brassica* polyploids [5]. The challenge to RNA-seq is that the transcripts of duplicated genes are difficult to precisely assign to homologous polyploids, especially in the absence of a reference genome [42]. MPV is a valid criterion to assess non-additive gene expression changes and functional plasticity in allopolyploids [43]. For RNA-seq, another shortcoming is that many short reads likely align to identical reference sequences, which may be excluded from transcript counting, thereby affecting the accuracy of estimated gene expression level [42]. Given the information mentioned above, both the DGE and non-additive genes identified in this study might not be as accurate as expected, and thus further verification is necessary.

**Table 2:** **Number of non-additively expressed genes in** ***Brassica*** **amphidiploids**

	**a**	**%**	**b**	**%**	**b/a(%)**	**c**	**%**	**c/a(%)**
	**No. of non-additively expressed genes Amphidiploid versus MPV**		**No. of non-additively expressed genes Amphidiploid > MPV**			**No. of non-additively expressed genes Amphidiploid < MPV**		
Bn	19844		9605		48.4	10239		51.6
Br > Bo	9519	48	5220	54.3	54.8	4299	42	45.2
Br < Bo	10325	52	4385	45.7	42.5	5940	58	57.5
Bj	20317		10240		50.4	10077		49.6
Br > Bg	11173	55	6429	62.8	57.5	4744	47.1	42.5
Br < Bg	9144	45	3811	37.2	41.7	5333	52.9	58.3
Bc	19921		7990		40	11931		60
Bg > Bo	9189	46.1	3399	42.5	37	5790	48.5	63
Bg < Bo	10732	53.9	4591	57.5	42.8	6141	51.5	57.2

## Discussion

### Differences in gene expression among *Brassica* diploids

Global *Brassica* research programs aim to explore valuable information on novel traits and to create stable lines. Br, Bg, and Bo exhibit many valuable agronomic traits including resistance against diseases and stress. These *Brassica* diploids have been suggested to have a triplication history [3]. Based on the DGE data of diploid *Brassica* species, multiple genes exhibited different expressional patterns in Br, Bg, and Bo. Moreover, 8932 genes were expressed in the leaf tissue of all three diploids. In addition, 2438, 2244, and 2029 genes were uniquely expressed in Br, Bg, and Bo, respectively. However, 5417 DEGs were differently expressed among *Brassica* diploids including genes that encode proteins with binding function, transmembrane transporter, glycosyltransferase (Bra013229 and Bra016237), acyltransferase (Bra018329, Bra018412, Bra033107, Bra037338, and Bra037725), and methyltransferase (Bra036774, Bra003928, Bra005371, Bra018386, and Bra021673). Different copies of homologs in A, B, and C *Brassica* genomes and a comparative mapping of *Brassica* have revealed extensive differences among the A, B, and C genomes [15,44]. The transcriptome changes in *Brassica* diploids are possibly due to the polyploid history during species formation. These changes cause different genome dosages and sub-functionalization/neo-functionalization of genes, as well as morphological/physiological differences in Br, Bg, and Bo. This result would facilitate the gene exploration related to species-specific characters, thereby accelerating the breeding of *Brassica*.

### Gene expression differences among allopolyploids and ancestral diploid progenitors

The expression differences in allotetraploids and diploids were analyzed by comparing the normalized value of genes expressed in each *Brassica* species. The results indicated that a larger number of gene expressional differences were identified between allotetraploids and diploids than among diploids. Although 11810 genes were conserved in Bn, Br, and Bo, 3102 DEGs were up-regulated in Bn compared with Br or Bo, and 1121 DEGs were down-regulated in Bn after natural polyploidization. Similarly, DEGs were also analyzed in Bj and Bc after polyploidization, and gene expressional changes were considered with parental preference. Zhao et al. (2013) also found that the gene expression in *Brassica* hexaploid is more similar to Br than to Bc [33]. In accordance with previous results, a large number of DEGs in natural Bn and Br/Bo suggests that the gene expression differences in resynthesized Bn might be effectively inherited after polyploidization [32,45,46]. These results indicated that long-term and immediate responses to polyploidization are complicated. Genome-biased expression and silencing of genes are also observed in natural and synthetic cotton [47]. Zhao et al. (2013) suggested that this observation might be due to the interactions of cytoplasm and nuclear genome during polyploidization [33]. Hitherto, Bj and Bc have been used for the creation of synthesized *Brassica* allopolyploids (AABBCC, AABC, BBAC, and CCAB) [48]. However, polyploidization of Bj and Bc have not been thoroughly studied. Given that the B genome possesses valuable agronomic traits including black-leg resistance [49], the study of B-genome evolution during the polyploidization of Bj and Bc is meaningful to the exploration of B-genome desirable traits. In the present research, many gene expressional differences in Bj and Bc after polyploidization were explored. The results showed that 5590 genes were differentially expressed in Bj, Br, and Bg, including genes that encode acyltransferase and metyltransferase. Moreover, the DEGs in Bj and Bc after polyploidization were more than that in Bn, which is partially due to the lack of a reference genome in this research. The B genome is more diversified than the A and C genomes [48]; hence, some B genome-specific information were neglected during the analysis of DGE data. Most of the commonly expressed genes in the diploids were non-additively expressed in allotetraploids, which is similar to the non-additive pattern in synthesized Bn and *Arabidopsis* [32,49]. The repression and activation of these genes in allotetraploids are responsible for the sub-functionalization of duplicated genes [47], which indicates a more complicated gene expression in allopolyploids rather than a simple combination of two genomes [46,48]. All of these non-additively expressed genes are important in studying the genome polyploidization of *Brassica*. The expressional changes in allotetraploids are necessary for the adjustment to the environment during natural polyploidization [33].

### Putative methyltransferase genes in *Brassica* allotetraploids

One of the epigenetic variations is DNA methylation, which is important to genome activity. Plant polyploidization is normally accompanied with various phenotypic changes that are partially induced by new methylation changes during the interaction of different genomes [50]. Extensive DNA methylation differences have been reported in synthesized Bn [45,51]. In the present research, 23, 36, and 33 methyltransferase genes were differentially expressed after the polyploidization of Bn, Bj, and Bc, respectively. The methyltransferase gene Bra020452 was up-regulated in Bn compared with Br and Bo, whereas the expression value of this gene in the early generations of resynthesized Bn was lower than that of natural Bn [32]. This finding implies the complexity of gene activation and silencing mechanism during *Brassica* polyploidization. Whether these methylation changes in *Brassica* are responsible for the different expressions of DEGs in allotetraploids needs to be verified. Further research of these genes is important to comprehend the transcriptome changes during *Brasssica* polyploidization.

## Conclusions

The genus *Brassica* includes a group of crops with important economic and nutritional values, and these crops are most closely related to *Arabidopsis. Brassica* and *Arabidopsis* have been confirmed to originate from a putative hexaploid ancestor. Triplication occurred after the divergence of *Brassica* and *Arabidopsis* to form a genomic complexity of *Brassica* [3]. Three allopolyploids, which arose from the natural hybridization of A, B, and C genomes, introduced extensive genome reshuffling and gene loss, as well as neo- and sub-functionalization of duplicate genes [6]. Therefore, the *Brassica* species are taken as an important model for the evolution of polyploids. Unfortunately, acknowledging the ancestors of *Brassica* polyploids is difficult, and these tetraploids are suspected to have multiple origins [52]. Resynthesizing *Brassica* allopolyploids have provided an alternative way to study polyploidization, but the research in this area mainly focused on *B. napus* [32]. An overview of the transcriptome differences among natural *Brassica* species would be interesting to gain initial knowledge on species diversification and polyploidization. This study demonstrated the DGE approach in characterizing the transcriptome of *Brassica* diploids and allotetraploids. However, the sampling from each genotype of *Brassica* may not capture the true range of phenotypes that exists within this genus. The DEGs during the evolution of *Brassica* diploids from a common hexaploid ancestor with *Arabidopsis* were revealed. Moreover, the DEGs in the natural polyploidization of *Brassica* allotetraploids from the hybridization of diploids were determined. Future work should concentrate on the function analysis of these DEGs, particularly stress resistance and methylase genes. Analysis should be performed to uncover the genetic and epigenetic mechanisms that would result in the phenotypic and physiologic differences among *Brassica* species. Elucidation of these differences is important to the discovery and utilization of genes for *Brassica* breeding and to shed light on *Brassica* evolution.

## Methods

### Plant materials

Diploid species *B. rapa* cv. Aikangqing (AA, 2*n* = 20), *B. nigra* cv. Marathi (BB, 2*n* = 16), and *B. oleracea* cv. Zhonghua Jielan (CC, 2*n* = 18) were used in the experiment. Amphidiploids *B. napus* cv. Yangyou 6 (AACC, 2*n* = 38), *B. juncea* cv. Luzhousileng (AABB, 2*n* = 36), and *B. carinata* cv. Dodolla (BBCC, 2*n* = 34) were also used as experimental materials. Plant materials were prepared and collected according to the procedures described by Kong et al. (2011) and Jiang et al. (2013) [32,53]. All plants were cultivated in climate chambers at 25°C, 16 h light/8 h dark photoperiod, and 70% relative humidity. The first true leaves from the three plants of each genotype were pooled at the same physiologic stage (28-day-old plants) and frozen at 80°C prior to use.

### RNA preparation, illumina RNA-sequencing, and data processing

Total RNA was extracted from the leaves by using an RNAiso Plus (Takara) according to the manufacturer’s protocol. RNA concentrations were measured using a Qubit fluorometer, and the integrity was confirmed using a 2100 Bioanalyzer (Agilent Technologies). DGE libraries were prepared using an Illumina Gene Expression Sample Prep Kit, and *Nla*III and *Mme*I were used for tag preparation. Single-chain molecules were fixed onto a Solexa sequencing chip (flowcell) and sequenced by an Illumina HiSeq™ 2000 System. Millions of raw 35 bp sequences were generated. Image analysis, base calling, generation of raw tags, and counting of tags were performed using the Illumina pipeline [34]. Empty tags (no tag sequence between the adaptors), adaptors, low-quality tags (tags containing one or more unknown nucleotides “N”), and tags with a copy number of 1 were removed from the raw sequences to obtain clean tags (21 bp) that contain CATG.

### Mapping of reads to the reference sequence

To identify the gene expression patterns in each genotype of *Brassica*, all clean tags were annotated by mapping onto the *B. rapa* genome [12] by using the SOAP2 software, and a maximum of one nucleotide mismatch is allowed [54]. All tags mapped to reference sequences were filtered, and the remaining tags were designated as ambiguous tags. Mapping events on sense and antisense sequences were included in the data processing. For gene expression analysis, the number of expressed tags was calculated and then normalized to the number of transcripts per million tags (TPM) [34,55]. The DEGs were screened and used for mapping and annotation [56,57]. Gene annotation was conducted using Blast2GO [58]. When the gene ontology (GO) database was searched, the GO categorization of all DEGs was displayed as three hierarchies for cellular component, molecular function, and biological process. Web gene ontology annotation plot was used to classify the genes mapped by each DGE library [59]. Clustering analysis of differential gene expression pattern was also conducted using a hierarchical clustering explorer [60,61]. In the present study, statistical comparison of the gene expression was performed according to the script written by Audic and Claverie [56]. False discovery rate (FDR) ≤ 0.001 and log2 ratio ≥1 were used as threshold to judge significance of gene expression difference [57]. Pathway enrichment analysis of differential gene expression was conducted to understand further the gene function through blasting the KEGG database. A *P*-value of 0.05 was selected as the threshold for considering a gene set as significantly enriched.

### Availability of supporting data

The sequence datasets that support the results of this article have been deposited in the Gene Expression Omnibus (GEO) at NCBI and are accessible under the accession GSE43246 (http://www.ncbi.nlm.nih.gov/geo/query/acc.cgi?acc=GSE43246).

## Additional files

Additional file 1:
**Distribution of total clean tags and distinct clean tags over different tag abundance categories in each DGE library.** (A) Distribution of total tags. Numbers in the brackets of indicate the range of copy numbers for a specific category of tags. For example, [2,5] means all the tags in this category has 2 to 5 copies. Numbers in the parentheses show the total tag copy number for all the tags in that category. (B) Distribution of distinct tags. Numbers in the square brackets indicate the range of copy numbers for a specific category of tags. Numbers in the parentheses show the total types of tags in that category. Additional file 2:
**Summary of tag mapping in DGE analysis for six libraries.**
Additional file 3:
**Mapping results of total tags and distinct tags of species in six libraries.** Normalized tag copy number was calculated by dividing tag counts for each gene with the total number of tags generated for each library and are presented per one million transcripts. PM and 1MM stand for perfect match and 1 miss match, respectively. (A) Mapping of total tags. (B) Mapping of distinct tags. Additional file 4:
**List of all** ***B. rapa*** **genes identified by DGE.** The first column represents names of the identified genes. Br_sense_raw and Br_antisense_raw mean the number of tags mapped to sense and antisense genes identified in DGE library of *B. rapa*. Br_sense_norm and Br_antisense_norm mean total times of detected tags per million in DGE library of *B. rapa*. GO Component, GO Function and GO Process mean the three main categories (cellular component, molecular function and biological process) in the GO classification, respectively. Additional file 5:
**List of all** ***A. thaliana*** **genes identified by DGE.** The first column represents names of the identified *Arabidopsis* genes. Br_raw means the number of DGE tags in *B. rapa* which were mapped to *A. thaliana* genome. Br_nom means total times of detected tags per million in DGE library of *B. rapa*. Additional file 6:
**Sequencing saturation analysis of the seven libraries of** ***B. rapa*** **(Br),** ***B. nigra*** **(Bg),** ***B. oleracea*** **(Bo),** ***B. napus*** (Bn), *B. juncea* (Bj), *B. carinata* (Bc). The number of detected genes was enhanced as the sequencing amount (total tag number) increased. Additional file 7:
**Distribution of ratio of distinct tag copy number in comparison of diploids and amphidiploids.** ‘A’ was the control and ‘B’ was experimental group in ‘A vs. B’. Additional file 8:
**List of differentially expressed genes and the top 20 most up-regulated and down-regulated genes between diploids and amphidiploids (‘A’ was the control and ‘B’ was experimental group in ‘A vs. B’).** TPM: transcript copies per million tags. Raw intensity: the total number of tags sequenced for each gene. FDR: false discovery rate. We used FDR < 0.001 and the absolute value of log2Ratio ≤1 as the threshold to judge the significance of gene expression difference. In order to calculate the log2Ratio and FDR, we used TPM value of 0.001 instead of 0 for genes that do not express in one sample. Additional file 9:
**List of genes for pathway enrichment analysis.** Pathways with Q value ≤0.05 are significantly enriched in DEGs, see red-border region (‘A’ was the control and ‘B’ was experimental group in ‘A vs. B’). Additional file 10:
**List of intersection DEGs used for HCE clustering analysis.**
Additional file 11:
**List of union DEGs used for HCE clustering analysis.**
Additional file 12:
**List of putative methyltransferase genes differentially expressed in** ***Brassica***.
