# Supplementary figures and images for: Digital gene expression analysis of gene expression differences within Brassica diploids and allopolyploids
Source: BMC Plant Biol. 2015 Jan 27;15:22. doi: 10.1186/s12870-015-0417-5 (PMC4312607; doi:10.1186/s12870-015-0417-5)

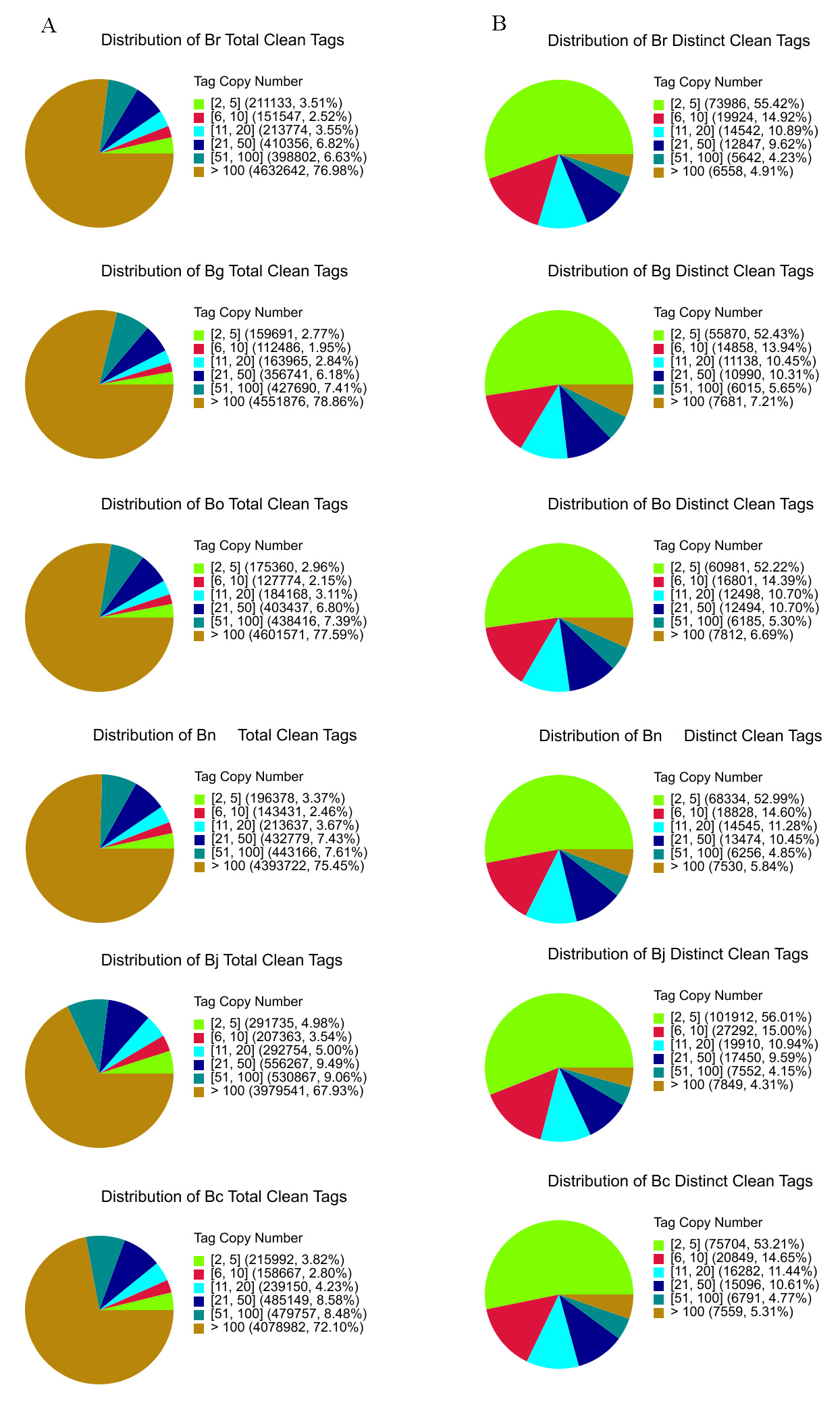

Supplement: Additional file 1: — Distribution of total clean tags and distinct clean tags over different tag abundance categories in each DGE library. (A) Distribution of total tags. Numbers in the brackets of indicate the range of copy numbers for a specific category of tags. For example, [2,5] means all the tags in this category has 2 to 5 copies. Numbers in the parentheses show the total tag copy number for all the tags in that category. (B) Distribution of distinct tags. Numbers in the square brackets indicate the range of copy numbers for a specific category of tags. Numbers in the parentheses show the total types of tags in that category. [file 12870_2015_417_MOESM1_ESM.jpeg]

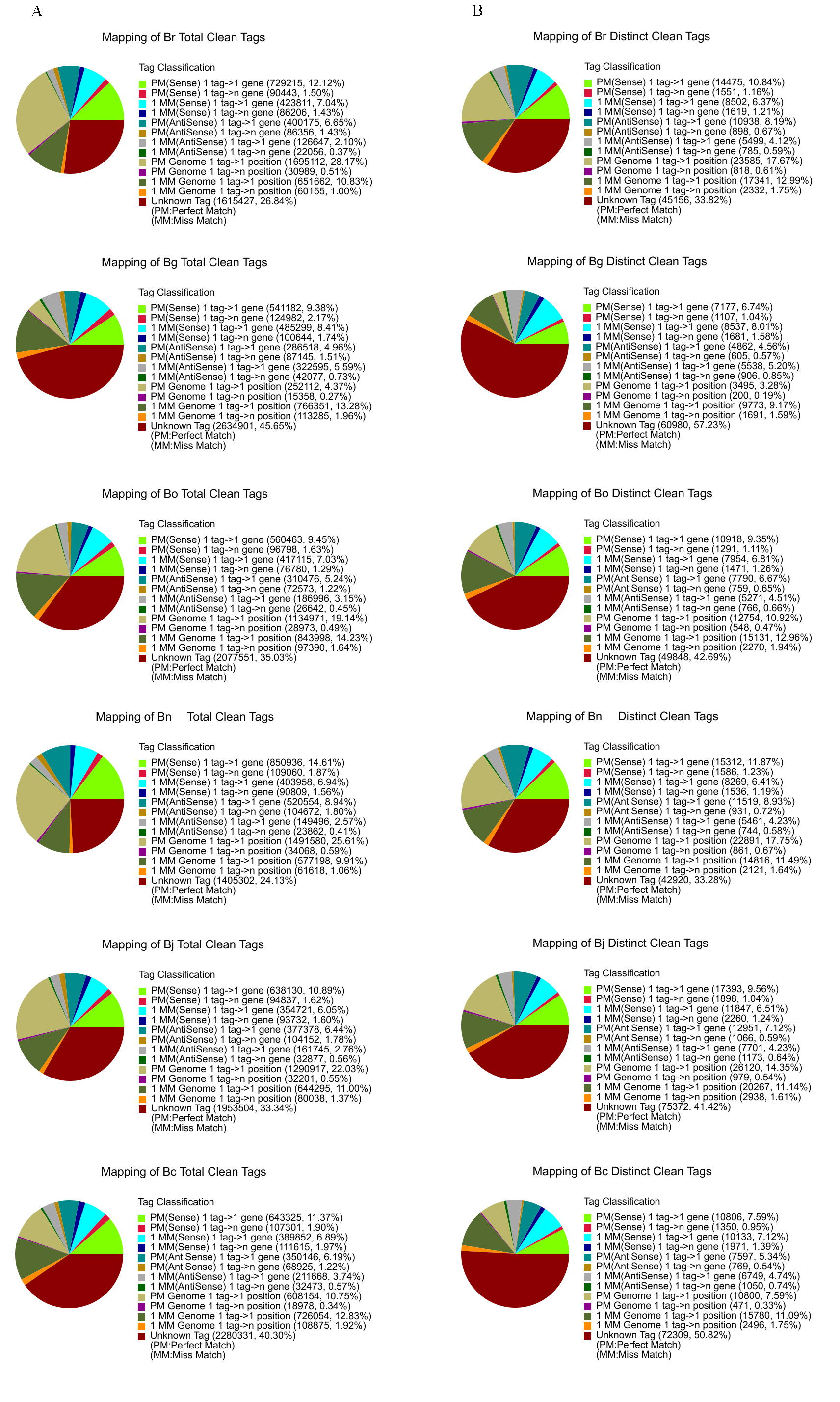

Supplement: Additional file 3: — Mapping results of total tags and distinct tags of species in six libraries. Normalized tag copy number was calculated by dividing tag counts for each gene with the total number of tags generated for each library and are presented per one million transcripts. PM and 1MM stand for perfect match and 1 miss match, respectively. (A) Mapping of total tags. (B) Mapping of distinct tags. [file 12870_2015_417_MOESM3_ESM.jpeg]

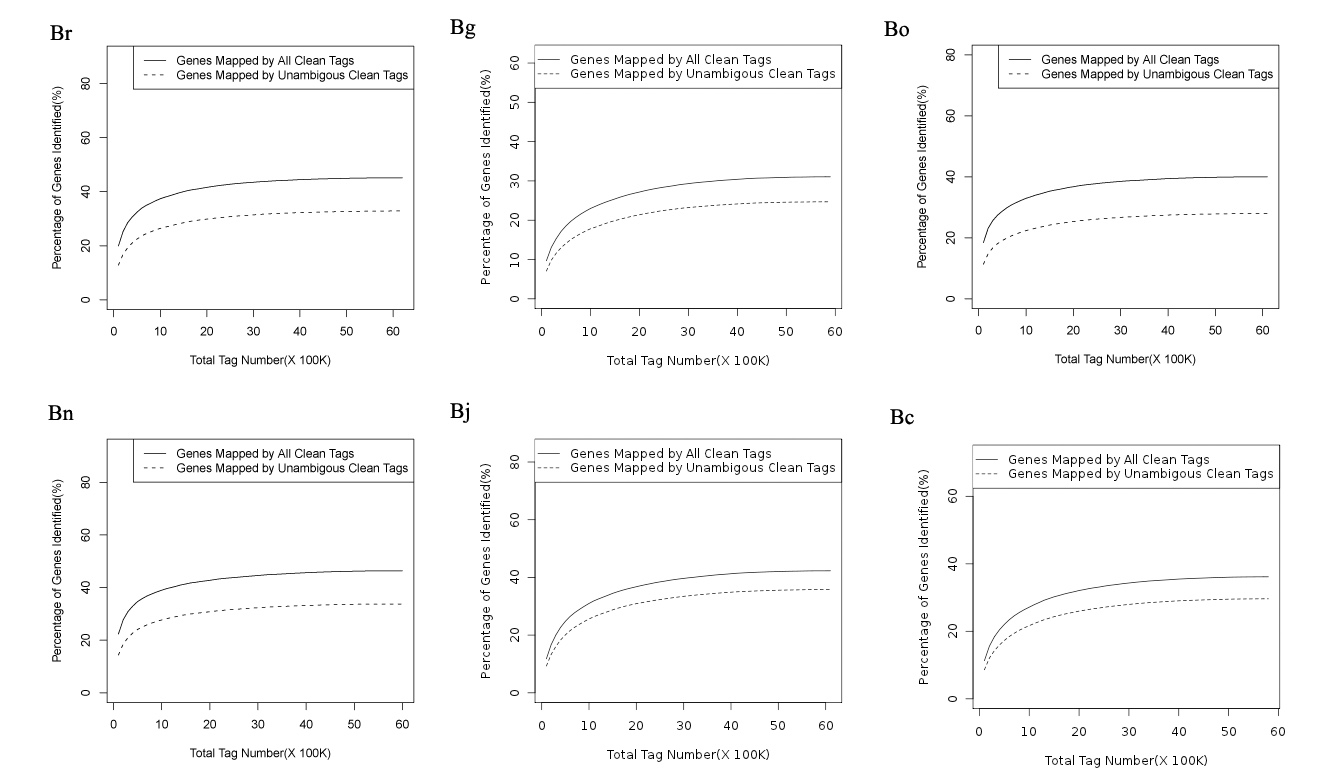

Supplement: Additional file 6: — Sequencing saturation analysis of the seven libraries of B. rapa (Br), B. nigra (Bg), B. oleracea (Bo), B. napus (Bn), B. juncea (Bj), B. carinata (Bc). The number of detected genes was enhanced as the sequencing amount (total tag number) increased. [file 12870_2015_417_MOESM6_ESM.jpeg]

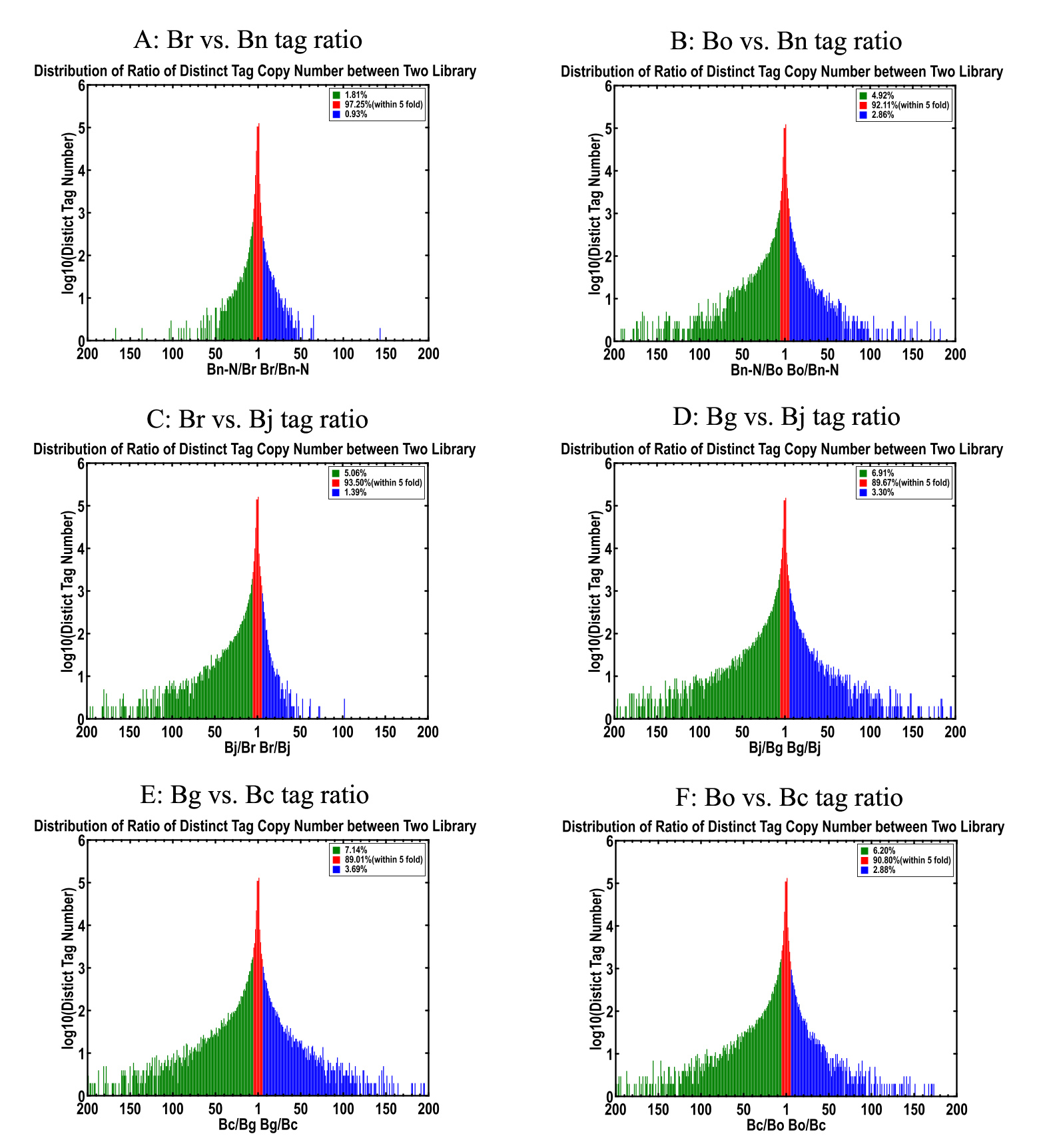

Supplement: Additional file 7: — Distribution of ratio of distinct tag copy number in comparison of diploids and amphidiploids. ‘A’ was the control and ‘B’ was experimental group in ‘A vs. B’. [file 12870_2015_417_MOESM7_ESM.jpeg]
